# Supplementary material for: Modeling glioblastoma heterogeneity as a dynamic network of cell states
Source: Mol Syst Biol. 2021 Sep 16;17(9):e10105. doi: 10.15252/msb.202010105 (PMC8444284; doi:10.15252/msb.202010105)
Supplement: Supplementary file 6 — Source Data for Figure 5 [file MSB-17-e10105-s004.zip › Figure5A_sourcedata/GSEA_3071/nosigdb_stateI.GseaPreranked.1622795145664/DOROSZKO_U3013MG_CLUSTER_1.html]

Details for gene set DOROSZKO\_U3013MG\_CLUSTER\_1[GSEA]

|  || Dataset | state13071 |
| Phenotype | NoPhenotypeAvailable |
| Upregulated in class | na\_pos |
| GeneSet | DOROSZKO\_U3013MG\_CLUSTER\_1 |
| Enrichment Score (ES) | 0.49495777 |
| Normalized Enrichment Score (NES) | 2.207155 |
| Nominal p-value | 0.0028248588 |
| FDR q-value | 5.3732865E-4 |
| FWER p-Value | 0.012 |
Table: GSEA Results Summary

  

Fig 1: Enrichment plot: DOROSZKO\_U3013MG\_CLUSTER\_1      
 Profile of the Running ES Score & Positions of GeneSet Members on the Rank Ordered List

  

| PROBE | GENE SYMBOL | GENE\_TITLE | RANK IN GENE LIST | RANK METRIC SCORE | RUNNING ES | CORE ENRICHMENT || 1 | SPP1 |  |  | 0 | 2.808 | 0.1153 | Yes |
| 2 | GJB2 |  |  | 8 | 1.906 | 0.1916 | Yes |
| 3 | NUPR1 |  |  | 21 | 1.585 | 0.2532 | Yes |
| 4 | POSTN |  |  | 32 | 1.494 | 0.3116 | Yes |
| 5 | BHLHE41 |  |  | 69 | 1.218 | 0.3513 | Yes |
| 6 | SERPINF1 |  |  | 71 | 1.211 | 0.4007 | Yes |
| 7 | NPC2 |  |  | 139 | 0.943 | 0.4202 | Yes |
| 8 | SNAI2 |  |  | 142 | 0.940 | 0.4582 | Yes |
| 9 | CHCHD10 |  |  | 225 | 0.778 | 0.4666 | Yes |
| 10 | AEBP1 |  |  | 236 | 0.761 | 0.4950 | Yes |
| 11 | LY96 |  |  | 451 | 0.547 | 0.4559 | No |
| 12 | PCDH7 |  |  | 478 | 0.529 | 0.4702 | No |
| 13 | GLMP |  |  | 583 | 0.464 | 0.4593 | No |
| 14 | BHLHE40 |  |  | 720 | 0.405 | 0.4368 | No |
| 15 | PIR |  |  | 950 | 0.339 | 0.3849 | No |
| 16 | GPNMB |  |  | 1070 | 0.312 | 0.3636 | No |
| 17 | SLC7A5 |  |  | 1084 | 0.310 | 0.3725 | No |
| 18 | FXYD5 |  |  | 1117 | 0.303 | 0.3758 | No |
| 19 | CEBPD |  |  | 1310 | 0.267 | 0.3316 | No |
| 20 | CD44 |  |  | 1430 | -0.251 | 0.3077 | No |
| 21 | CHCHD6 |  |  | 1473 | -0.254 | 0.3061 | No |
| 22 | FARP1 |  |  | 1496 | -0.257 | 0.3103 | No |
| 23 | ENAH |  |  | 1599 | -0.267 | 0.2919 | No |
| 24 | EDNRB |  |  | 1754 | -0.287 | 0.2595 | No |
| 25 | STK32A |  |  | 1790 | -0.292 | 0.2614 | No |
| 26 | TRIM2 |  |  | 1803 | -0.294 | 0.2700 | No |
| 27 | ZNF106 |  |  | 2487 | -0.410 | 0.0905 | No |
| 28 | GMPR |  |  | 2516 | -0.417 | 0.0996 | No |
| 29 | PLEKHA2 |  |  | 3049 | -0.634 | -0.0273 | No |
| 30 | LZTS1 |  |  | 3052 | -0.636 | -0.0017 | No |
| 31 | AP1S2 |  |  | 3206 | -0.789 | -0.0133 | No |
| 32 | ETV5 |  |  | 3364 | -1.078 | -0.0142 | No |
| 33 | NBL1 |  |  | 3432 | -1.368 | 0.0227 | No |
Table: GSEA details [plain text format]

  

Fig 2: DOROSZKO\_U3013MG\_CLUSTER\_1: Random ES distribution      
 Gene set null distribution of ES for **DOROSZKO\_U3013MG\_CLUSTER\_1**
